# Prediction and Screening of Lead-Free Double Perovskite Photovoltaic Materials Based on Machine Learning

**DOI:** 10.3390/molecules30112378

**Published:** 2025-05-29

**Authors:** Juan Wang, Yizhe Wang, Xiaoqin Liu, Xinzhong Wang

**Affiliations:** Xi’an Key Laboratory of Advanced Photo-Electronics Materials and Energy Conversion Device, School of Electronic Information, Xijing University, Xi’an 710123, China

**Keywords:** double perovskite, lead free and non-toxic, XGBoost model, bandgap prediction, material screening

## Abstract

The search for stable, lead-free perovskite materials is critical for developing efficient and environmentally friendly energy solutions. In this study, machine learning methods were applied to predict the bandgap and formation energy of double perovskites, aiming to identify promising photovoltaic candidates. A dataset of 1053 double perovskites was extracted from the Materials Project database, with 50 feature descriptors generated. Feature selection was carried out using Pearson correlation and mRMR methods, and 23 key features for bandgap prediction and 18 key features for formation energy prediction were determined. Four algorithms, including gradient-boosting regression (GBR), random forest regression (RFR), LightGBM, and XGBoost, were evaluated, with XGBoost demonstrating the best performance (R^2^ = 0.934 for bandgap, R^2^ = 0.959 for formation energy; MAE = 0.211 eV and 0.013 eV/atom). The SHAP (Shapley Additive Explanations) analysis revealed that the X-site electron affinity positively influences the bandgap, while the B″-site first and third ionization energies exhibit strong negative effects. Formation energy is primarily governed by the X-site first ionization energy and the electronegativities of the B′ and B″ sites. To identify optimal photovoltaic materials, 4573 charge-neutral double perovskites were generated via elemental substitution, with 2054 structurally stable candidates selected using tolerance and octahedral factors. The XGBoost model predicted bandgaps, yielding 99 lead-free double perovskites with ideal bandgaps (1.3~1.4 eV). Among them, four candidates are known compounds according to the Materials Project database, namely Ca_2_NbFeO_6_, Ca_2_FeTaO_6_, La_2_CrFeO_6_, and Cs_2_YAgBr_6_, while the remaining 95 candidate perovskites are unknown compounds. Notably, X-site elements (Se, S, O, C) and B″-site elements (Pd, Ir, Fe, Ta, Pt, Cu) favor narrow bandgap formation. These findings provide valuable guidance for designing high-performance, non-toxic photovoltaic materials.

## 1. Introduction

Metal halide perovskite materials have been widely applied in fields such as solar cells, photodetectors, and photocatalysis due to their large optical absorption coefficient, bipolar charge transport characteristics, long carrier diffusion length, low exciton binding energy, and other advantages [1,2]. However, the presence of issues such as poor stability, weak tunability, and lead pollution has restricted their commercial applications [3,4]. Against the backdrop of the current advocacy for the development of efficient and environmentally friendly energy, finding perovskite materials that are stable, that have tunable optoelectronic properties, and are free of lead toxicity is a hot issue that urgently needs to be addressed. Researchers have obtained a double perovskite structure by replacing two toxic Pb^2+^ ions in the single perovskite lattice with a pair of non-toxic monovalent and trivalent metal cations [5,6]. Lead-free double perovskites possess characteristics such as environmental friendliness, super stability, and tunable optoelectronic properties, showing broad application prospects in the photovoltaic field and other areas [7]. They are expected to replace lead-based halide perovskites and become the next generation of high-efficiency and stable optoelectronic materials.

The chemical compositions and structures of double perovskite materials are complex and diverse. Traditional experimental trial-and-error methods and density functional theory (DFT) calculation methods are time consuming and labor intensive, and it is difficult to meet the demand for rapidly screening candidate material compounds. Artificial intelligence has shown great potential in the field of materials science. It has been proven that machine learning can accelerate the discovery process of new materials by analyzing a large amount of data and identifying patterns, and it has been widely applied to the prediction and screening of perovskite materials [8,9], which can significantly improve the efficiency of material design [10].

The bandgap determines the optoelectronic properties of perovskite materials [11]. By predicting the bandgap value of perovskite compounds, it can help to screen the candidate perovskite compounds in the specific bandgap range and accelerate the discovery of perovskite materials. Lu et al. [12] proposed a rapid target-driven method that combines machine learning and density functional theory calculation. They trained a machine learning model using 212 known perovskite structures and successfully predicted the bandgap values of 5158 perovskites. Zhan et al. [13] utilized the various machine learning algorithms to compare the predictive performance of different models, and revealed the crucial factors that influence the bandgap properties of perovskite materials. Ghosh et al. [14] used four supervised machine learning models to predict the bandgap of calcium nitride perovskite compounds, and it was shown that the random forest regression (RFR) algorithm performed the best. Sradhasagar et al. [15] used a machine learning model to prepare a list of novel bismuth-based double perovskite oxides with predicted bandgap values and types from a vast chemical space. Guo et al. [16] used multiple machine learning methods to predict the bandgap of lead-free halide double perovskites, and it was shown that the random forest was the best for bandgap prediction. Gao et al. [17] proposed a novel search strategy that combines machine learning and DFT calculation, and screened out two novel lead-free inorganic double perovskites from 5796 inorganic double perovskites and gave the direct bandgap values. Luo et al. [18] introduced an improved parallel residual network (PRN), using atomic composition as input data to predict the bandgap of lead-free inorganic double perovskite materials, showing superior prediction accuracy. These studies demonstrate the great potential of machine learning in the bandgap prediction of various perovskite materials.

The key indicator for the synthesizability of perovskite materials is the formation energy. The larger the negative value of the formation energy, the easier it is for the compound to form and the higher its thermodynamic stability [19]. Significant progress has also been made in the application of machine learning in predicting the formation energy of perovskite compounds, which can assist in the screening and design of perovskite materials [20]. Chen et al. [21] used three machine learning methods, namely extreme trees, gradient-boosting decision trees, and multi-layer perceptrons, combined with improved elemental property matrix descriptors, to predict the formation energy of cubic perovskites. Among them, the R^2^ score reached up to 97%, which is a significant improvement compared to traditional descriptors and provides an important reference for the rapid prediction of perovskite materials. Choubisa et al. [22] proposed a crystal site feature embedded in a convolutional neural network (CNN) and an extended deep neural network (EDNN) to accelerate the prediction of the formation energy of halide perovskites with a relatively low mean absolute error. Deringer et al. [23] demonstrated the effectiveness of gaussian process regression in predicting the formation energy of compounds, showing the potential of machine learning in accelerating material discovery. Zhang et al. [24] used machine learning algorithms, taking elemental oxidation states and electronegativities as input features, to predict the formation energy of perovskites. They found that both the XGBoost and LightGBM models performed better than the random forest method, and at the same time, they helped to quickly screen out three potential perovskite candidates for photovoltaic applications.

Although machine learning has shown great potential in the prediction and screening of perovskite materials, there are still many challenges in the research of prediction, screening, and design of double perovskites. Firstly, the performance of machine learning models is largely limited by the quality and quantity of existing double perovskite training data, and the scale and diversity of the existing datasets are still insufficient to meet the ideal requirements. Secondly, the machine learning models for double perovskite materials lack interpretability [25]. Thirdly, the hidden relationships between the structure and properties of perovskite materials still need further exploration. Therefore, in-depth research on double perovskite materials through machine learning is still required to provide more scientific reference value and promote the design and application of perovskite materials.

In this study, four machine learning models, namely gradient-boosting regression (GBR), random forest regression (RFR), XGBoost, and LightGBM, were used to predict the bandgap and formation energy of lead-free double perovskite materials. The SHAP (Shapley Additive Explanations) method was employed to interpret the prediction results of the models, revealing the hidden relationships between structural features and properties. Moreover, a large number of virtual double perovskite structures were generated through the element substitution method. Combined with the optimized XGBoost model, 99 kinds of lead-free double perovskite candidate materials with a narrow bandgap (1.3~1.4 eV) suitable for photovoltaic applications were screened out, providing a reference for the design of photovoltaic materials.

## 2. Results and Discussion

### 2.1. Data Preprocessing

Data preprocessing of the dataset was carried out through exploratory data analysis. MinMaxScaler was adopted to normalize the original data to ensure that all features were within the same scale range, thereby improving the training effect and prediction accuracy of the model. The working principle of MinMaxScaler is as follows:

(1) Find the minimum value, min, and the maximum value, max, of each feature in the training set.
(2) For each sample, calculate the normalized result of its feature value, as shown in formula (1).
  (1) $$X_{normalize}=(x-min)/(max-min)$$
(3) Map the value of each feature to the interval of [0, 1].

Box plots were used for visual analysis of the data. From the box plots of the 50 pieces of original feature data (Figure S1), outliers in the data can be observed. If there are obvious outliers, that is, significantly abnormal values, in the feature data, these abnormal data should be considered for deletion during feature processing.

### 2.2. Feature Extraction and Selection

Feature selection is a technique that reduces data dimensionality and improves model performance by selecting the subset of features that are most relevant to the target variable, which can enhance the accuracy and generalizability of the model.

The Pearson correlation coefficient, a statistic used to measure the strength of the linear relationship between two variables, is often employed to evaluate the correlation between features and the target variable, aiding in the determination of the most valuable feature subset. In this paper, the Pearson correlation coefficient is used to measure the degree of linear correlation between two features and between a feature and the target variable, and its definition is shown in Formula (2).

(2) $$\rho_{X,Y}=\frac{cov(X,Y)=1/(n-1)\sum_{n}^{i=1}(X_{i}-X^{-})(Y_{i}-Y^{-})}{\sigma_{X}\sigma_{Y}}$$

The Pearson correlation coefficient ranges from −1 to 1, where −1 indicates a perfect negative correlation, 1 indicates a perfect positive correlation, and 0 indicates no linear correlation. The sign represents the direction of the correlation, and the magnitude of the value represents the degree of correlation. If the Pearson correlation coefficient between two features is too large, this indicates that there is redundancy between the features. The screened features are further ranked through the Minimum Redundancy Maximum Relevance (mRMR) method. If the Pearson correlation coefficient between two features is greater than 0.90, the feature with a lower ranking according to mRMR is deleted.

**Figure 1:**
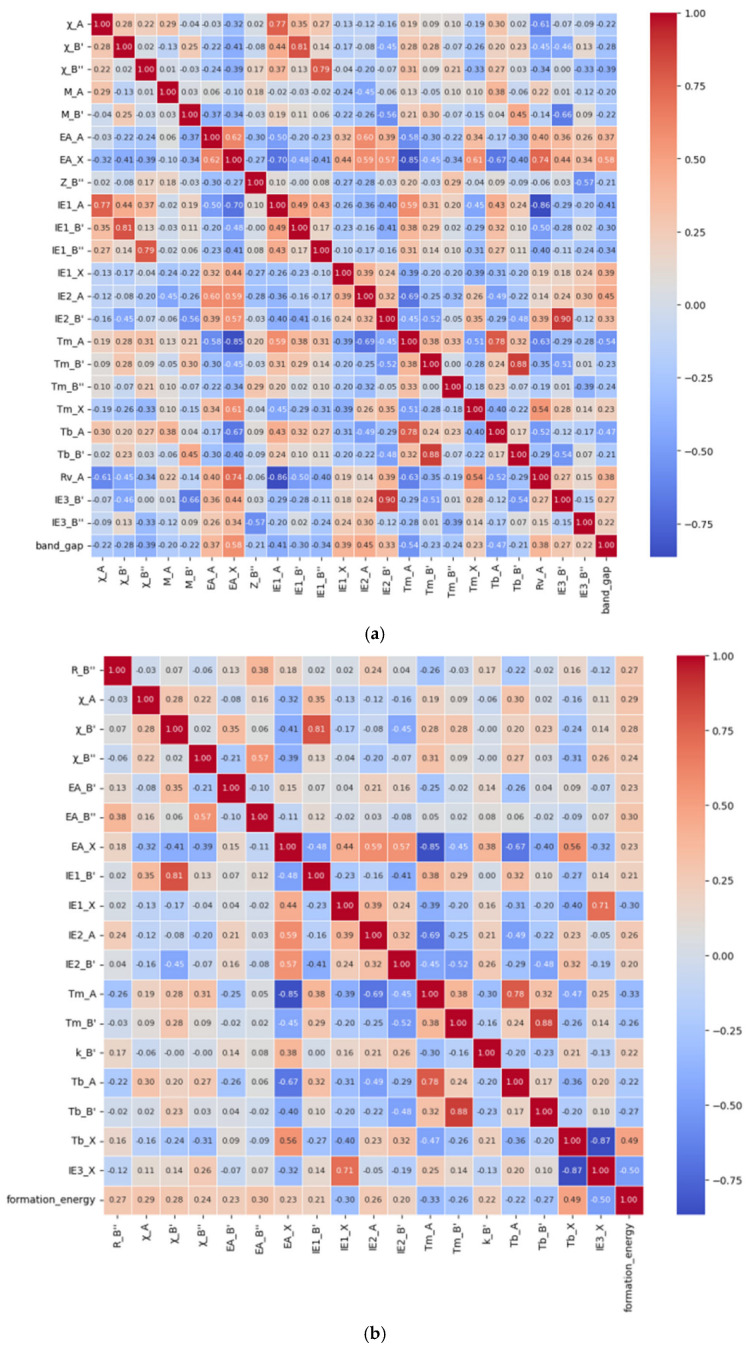
The Pearson correlation diagram of bandgap (**a**) and formation energy (**b**).

In the bandgap prediction, a total of 23 relevant features, namely the electronegativity of the A-site atom, B′-site atom, and B″-site atom (χ_A, χ_B′ and χ_B″), atomic mass of the A-site and B′-site elements (M_A and M_B′), electronic affinity of the A-site and X-site elements (EA_A and EA_X), atomic number of the B″-site element (Z_B″), first ionization energy of the A-site, B′-site, B″-site, and X-site elements (IE1_A, IE1_B′, IE1_B″ and IE1_X), second ionization energy of the A-site and B′-site elements (IE2_A and IE2_B′), melting-point temperature of the A-site, B′-site, B″-site, and X-site elements (Tm_A, Tm_B′, Tm_B″ and Tm_X), boiling-point temperature of the A-site and B′-site elements (Tb_A and Tb_B′), van der Waals radius of the A-site element (Rv_A), and third ionization energy of the B′-site and B″-site elements (IE3_B′ and IE3_B″) were finally retained, and the Pearson correlation diagram of these features was drawn, as shown in Figure 1a. Similarly, in the formation energy prediction, 18 relevant features, namely the B″-site ionic radius (R_B″), electronegativity of the A-site, B′-site and B″-site atoms (χ_A, χ_B′ and χ_B″), electronic affinity of the B′-site, B″-site, and X-site elements (EA_B′, EA_B″ and EA_X), first ionization energy of the B′-site and X-site elements (IE1_B′ and IE1_X), second ionization energy of the A-site and B′-site elements (IE2_A and IE2_B′), melting-point temperature of the A-site and B′-site elements (Tm_A and Tm_B′), thermal conductivity of the B′-site element (k_B′), boiling-point temperature of the A-site, B′-site, and X-site elements (Tb_A, Tb_B′ and Tb_X), and third ionization energy of the X-site element (IE3_X) were finally retained, and the Pearson correlation diagram of these features was drawn, as shown in Figure 1b. The gradient color bar on the right corresponds to the magnitude of the correlation coefficient. Red indicates a positive correlation and blue indicates a negative correlation, and the lighter the color, the lower the correlation.

In order to optimize the performance of the model and reduce the redundancy of feature, four regression models (GBR, RFR, XGBoost, and LightGBM) were used for feature selection. By gradually removing the features with lower importance, the changing trends in the number of feature subsets and the performance indicators (R^2^ and adjusted R^2^) of the model were recorded. In the feature number scoring diagrams (Figures S2 and S3) for bandgap and formation energy prediction, it was found that the R^2^ value of the XGBoost model was the largest. By removing the features with lower importance, the optimal feature subset for bandgap prediction was determined to be χ_B′, χ_B″, M_A, M_B′, EA_X, Z_B″, IE1_B′, IE1_B″, IE1_X, IE2_A, IE2_B′, Tm_B′, Tm_B″, Tb_B′, IE3_B′, and IE3_B′. The optimal feature subset for formation energy prediction was χ_A, χ_B′, χ_B″, EA_X, IE1_X, and IE2_A.

### 2.3. Establishment of the Prediction Model

**Figure 2:**
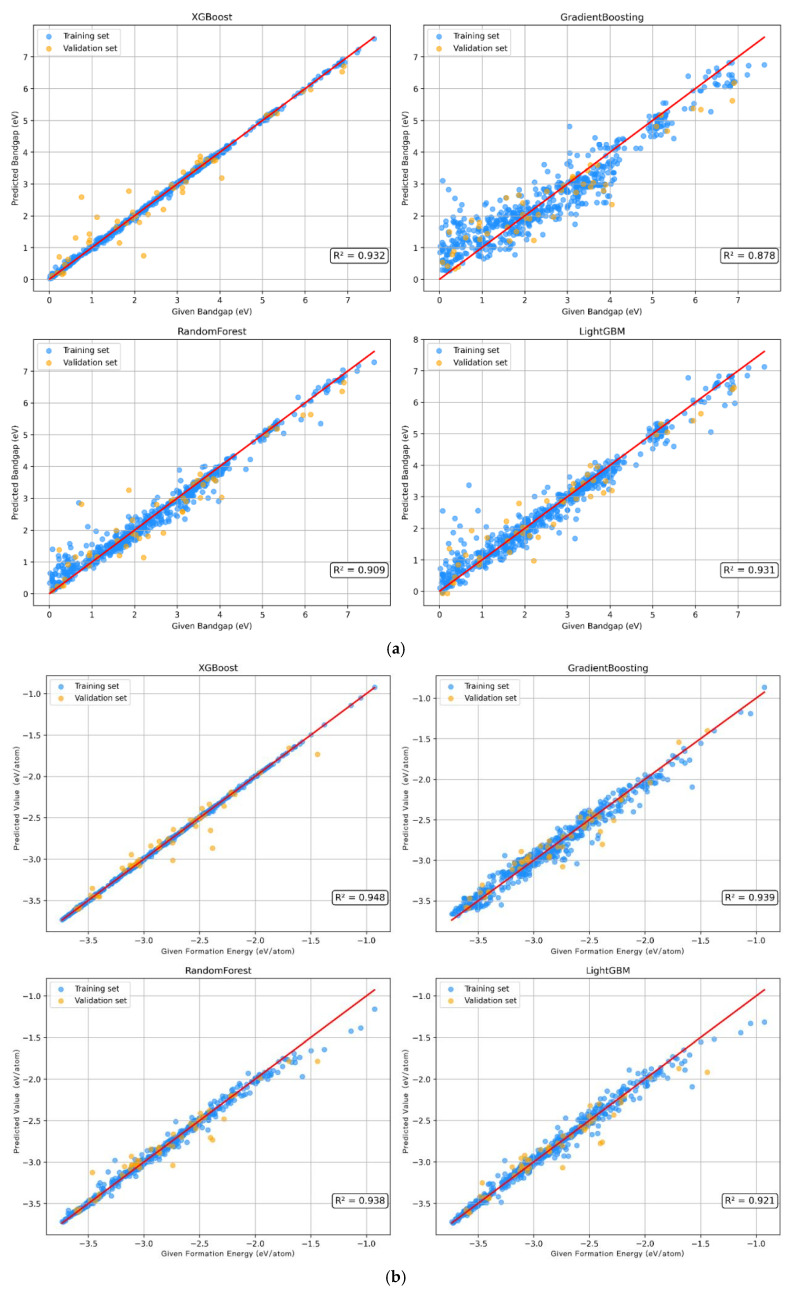
The bandgap (**a**) and formation energy (**b**) prediction based on machine learning algorithms.

A total of 15% of the data in the original dataset was reserved as the test set, and the remaining data were divided into the training set and the validation set at a ratio of 0.85:0.15. The performance of the four models in predicting the bandgap was compared. Through the comprehensive evaluation of R^2^, MSE, and MAE, XGBoost has the best performance in terms of model fit and prediction error. Table 1 shows that the R^2^ value of XGBoost is 0.932, MSE is 0.218, and MAE is 0.297, indicating their strong generalizability. As can be seen from the fitting effect in Figure 2a, both the training set and the validation set of the XGBoost model perform well, and the distribution of data points is similar. It is indicated that its prediction effect on this dataset is excellent, and it has strong fitting ability and generalizability. In order to verify the performance of the model, 10-fold cross validation was used to evaluate each model (Table S1). The results also show that the XGBoost model performs the most stably among all these four models, with strong generalizability and small errors.

Similarly, the above four models were used to predict the formation energy. Table 1 shows that according to the three indicators of R^2^, MSE, and MAE, the XGBoost model performed the best. And, the XGBoost model has the smallest prediction error for the formation energy. According to the fitting effect of each model (Figure 2b), it shows that the XGBoost model exhibits the best fitting effect and generalizability among the four models. The 10-fold cross-validation results (Table S2) show that the distribution of R^2^ values of the XGBoost model is more concentrated, which means that this model has strong generalizability and shows consistent performance under different data folds.

In order to further improve the stability of the model, reduce overfitting, and improve the generalizability of the model, the key hyperparameters of the XGBoost model were optimized by using the grid search method, and the combination with the best performance according to the XGBoost model in the cross validation was selected (Table S3) to maximize the prediction performance of the model.

### 2.4. Evaluation of the Prediction Model

In order to test the model′s ability to predict unknown data, 15% of the samples reserved in advance were used as the test set. The prediction results of the bandgap and formation energy of perovskites in the test set by the XGBoost model are shown in Table 2. The MAE and RMSE values of the bandgap prediction model are 0.211 eV and 0.259 eV, respectively. Compared with the variation range of 0 to 6 eV, the errors are within an acceptable range. The R^2^ values of the bandgap and formation energy predictions are 0.934 and 0.959, respectively, indicating that the model has strong generalizability and can perform well on the test set.

### 2.5. Analysis of Model Prediction Results Using the SHAP Method

The SHAP (Shapley Additive Explanations) method is helpful for model interpretation and feature selection. It can also assist in verifying the correctness and reliability of the model, identifying the weaknesses of the model, and proposing improvement plans. Through the SHAP model interpretation diagram, one can understand the contribution of each feature to the model prediction result, as well as the direction and degree of influence of the feature values.

**Figure 3:**
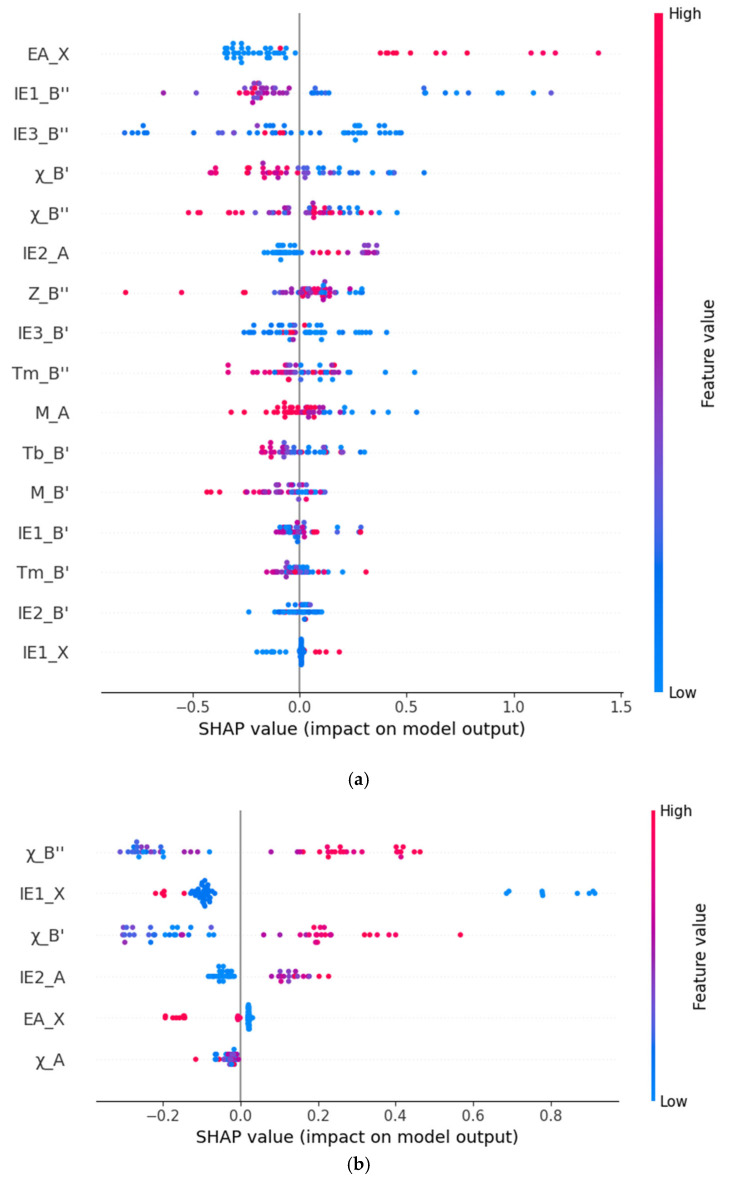
Scatter plots of feature importance for predicting bandgap (**a**) and formation energy (**b**) by the XGBoost Model.

Figure 3a shows the SHAP values of the top 16 most relevant features in the bandgap prediction model. The horizontal axis represents the average SHAP value of the features (that is, the average contribution of the features to the model output), and each bar represents the degree of influence of the feature on the prediction of the formation energy. The larger the SHAP value, the greater the contribution of the feature to the model prediction result. According to the magnitude of the SHAP values of the features, the importance of the features is arranged in descending order. Red indicates a positive influence, that is, the higher the value of this feature, the stronger the positive correlation between the predicted value and the actual value; blue indicates a negative influence, that is, the higher the value of this feature, the stronger the negative correlation between the predicted value and the actual value. Through analysis, it can be seen that the electron affinity of the element at the X position (EA_X), the first ionization energy of the element at the B″ position (IE1_B″), and the third ionization energy of the element at the B″ position (IE3_B″) are the three most important features in the model. The feature of the electron affinity of the element at the X position (EA_X) has a positive correlation with the predicted bandgap value. The two features, the first ionization energy of the element at the B″ position (IE1_B″) and the third ionization energy of the element at the B″ position (IE3_B″), have a negative correlation with the predicted bandgap value. Secondly, the feature of the electronegativity of the atom at the B′ position (χ_B′) also shows an obvious negative correlation with the predicted bandgap value.

Similarly, in Figure 3b, the importance ranking of the features related to the formation energy prediction is shown. It can be seen that the first ionization energy of the element at the X position (IE1_X), the electronegativity of the atom at the B″ position (χ_B″), and the electronegativity of the atom at the B′ position (χ_B′) are the three most important features for the formation energy prediction. Among them, the first ionization energy of the element at the X position (IE1_X) has a negative correlation with the predicted formation energy value, and the electronegativities of the atoms at the B″ position (χ_B″) and the B′ position (χ_B′) have a positive correlation with the predicted formation energy value.

### 2.6. Lead-Free Double Perovskite Screening for Photovoltaic Materials

#### 2.6.1. Sample Generation

**Figure 4:**
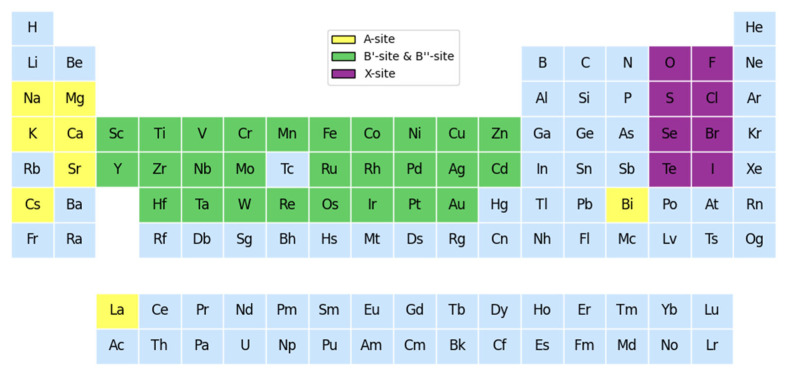
The selection of elements at each site according to the method of element substitution.

The method of element substitution was adopted to fill different elements at the A, B′, B″, and X positions, including 8 class A cations, 27 class B′ cations, 27 class B″ cations, and 8 class X anions from the original dataset, as shown in Figure 4. A total of 4573 electrically neutral lead-free double perovskite structures were generated and their key features were filled in to form a predictive dataset. According to the ionic radius of the elements at the four sites, the tolerance factor (T_f_) and octahedral factor (O_f_) corresponding to each structure were calculated. The optimized XGBoost was used to predict the bandgap and formation energy of these candidate materials.

#### 2.6.2. Screening of Virtual Samples

Firstly, it is determined whether the candidate materials can form a stable perovskite structure according to the T_f_ (tolerance factor) and O_f_ (octahedral factor) values. The ideal range of the T_f_ value is from 0.85 to 1.05, and the Of value should be between 0.4 and 0.7 to ensure the octahedral stability of the structure. Therefore, the screening conditions are set as 0.85 < T_f_ < 1.05 and 0.4 < O_f_ < 0.7, and the number of samples is reduced to 2054. In order to ensure the rationality of the screening, the calculations of the tolerance factor (T_f_) and the octahedral factor (O_f_) are based on the ionic radii of the candidate materials, and the formulas are as follows:

(3) $$T_{f}=\frac{r_{A}+r_{X}}{\sqrt{2}\cdot \frac{\left(r_{B^{\prime }}+r_{B^{\prime \prime }}\right)}{2}+r_{X}}$$

(4) $$O_{f}=\frac{\frac{\left(r_{B^{\prime }}+r_{B^{\prime \prime }}\right)}{2}}{r_{X}}$$

Among them, $r_{B^{\prime }}$ and $r_{B^{\prime \prime }}$ are the ionic radii of the cations at the B′ and B″ positions, and $r_{X}$ is the ionic radius of the anion at the X position. The octahedral factor reflects the geometric stability between the B-site cations and the X-site anions. By calculating these geometric parameters, candidate double perovskite structures with geometric stability can be effectively screened out, thus improving the accuracy of subsequent bandgap predictions.

According to the bandgap values predicted by the XGBoost model, materials with a bandgap in the range of 1.00~1.60 eV were selected as photovoltaic candidate materials, and 670 combinations were successfully screened out. For high-performance photovoltaic materials, the suitable bandgap value of double perovskites is usually 1.1~1.4 eV [26]. Also, according to the Shockley–Queisser [27] limit, the ideal optical absorption bandgap of a single-junction solar cell is 1.34 eV. Therefore, the range of the bandgap was further narrowed down to 1.3~1.4 eV, and 99 double perovskite materials that are more likely to become photovoltaic materials were screened out (Table S4). Among them, four candidates are known compounds according to Materials Project database, namely Ca_2_NbFeO_6_, Ca_2_FeTaO_6_, La_2_CrFeO_6_, and Cs_2_YAgBr_6_, while the remaining 95 candidate perovskites are unknown compounds.

#### 2.6.3. Sample Analysis

**Figure 5:**
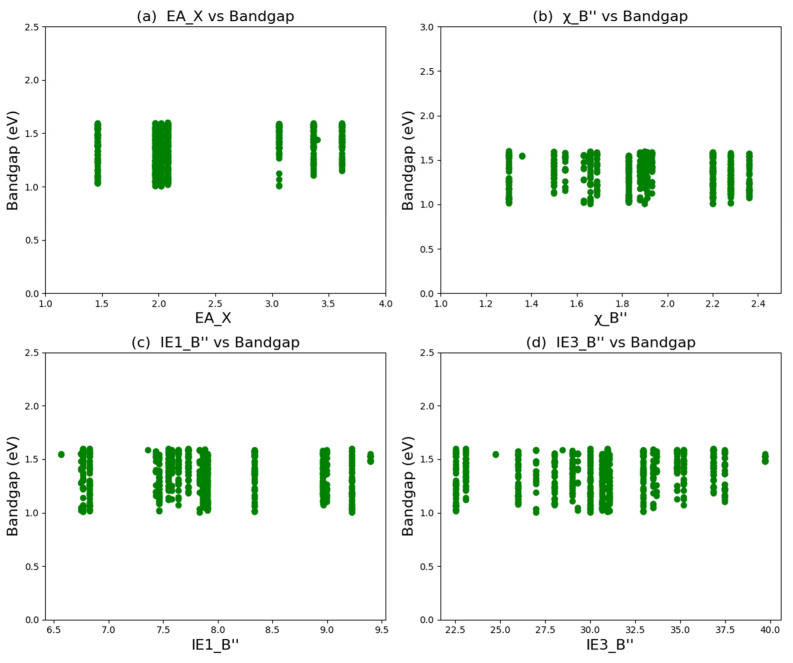
Statistical analysis of bandgap prediction data of 670 double perovskite materials: (**a**) electron affinity of elements at the X position; (**b**) electronegativity of elements at the B″ position; (**c**) first ionization energy of elements at the B″ position; (**d**) third ionization energy of elements at the B″ position.

In order to reveal the hidden trends in the data of double perovskite materials, data analysis was carried out on the bandgap prediction set (670 perovskites with a bandgap in the range of 1.00~1.60 eV). According to the analysis of the characteristic variables by the SHAP method, the electron affinity of the element at the X position, the electronegativity of the element at the B″ position, and the first ionization energy of the element at the B″ position play the most important roles in the bandgap of double perovskites, followed by the third ionization energy of the element at the B″ position. Statistical analysis was conducted on these four characteristic variables, and the results are shown in Figure 5. The elements with the highest frequencies at the X position are Se, S, O, and Cl, and the elements with the highest frequencies at the B″ position are Pd, Ir, Fe, Ta, Pt, and Cu. When the element at the A position is one of these four elements and the element at the B″ position is one of these six elements, it is more likely to form a narrow-bandgap double perovskite. These findings can provide some reasonable design strategies for the design of double perovskites with potential photovoltaic applications.

## 3. Data and Methods

### 3.1. Data Source

**Figure 6:**
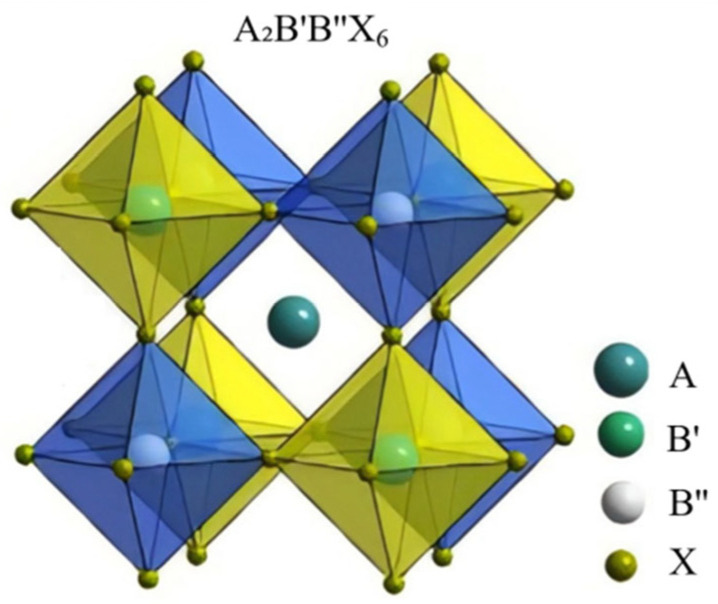
Schematic diagram of the structure of double perovskite material.

The dataset for the prediction of the double perovskite structure and formation energy includes 1053 pieces of original data. These original data are sourced from the Materials Project, an open-access materials database developed by Carnegie Mellon University and Lawrence Berkeley National Laboratory. Its main objective is to accelerate material discovery and the design of new materials. The structure of the double perovskite is shown in Figure 6. The predicted characteristic descriptors include a total of 50 features such as the ionic radius information of the elements at the A, B′, B″, and X positions in their respective valence states, the tolerance factor, and the octahedral factor, as well as the physical and chemical properties of the elements (Table S5).

### 3.2. Machine Learning Algorithms and Model Evaluation

#### 3.2.1. Gradient-Boosting Regression (GBR)

Gradient-boosting regression (GBR) [28] obtains more accurate prediction results by gradually constructing multiple weak learners and combining them into a strong learner. Its core idea is to gradually reduce the residuals of the model by optimizing the loss function.

#### 3.2.2. Random Forest Regression

Random forest regression [29] improves the prediction accuracy and stability of regression tasks by constructing multiple decision trees and averaging the prediction results of these decision trees. Its core idea is to construct multiple decision trees by introducing randomness, and then obtain the final prediction result by averaging the prediction results of these trees.

#### 3.2.3. Extreme Gradient Boosting (XGBoost)

XGBoost (eXtreme Gradient Boosting) [30] constructs a strong learner by combining multiple weak learners (usually decision trees). Its core idea is to gradually add new tree models, and each tree attempts to correct the prediction error of the previous tree, thereby gradually improving the prediction accuracy of the model.

The objective function of XGBoost consists of two parts: the loss function and the regularization term. The loss function measures the error between the model′s predicted values and the true values, while the regularization term is used to control the complexity of the model and prevent overfitting. The objective function can be expressed as follows:

(5) $${obj}^{\left(t\right)}=\sum_{i=1}^{n}l\left(y_{i},{y_{\begin{array}{c} i \end{array}}}^{\left(t-1\right)}+f_{t}\left(x_{i}\right)\right)+\Omega \left(f_{t}\right)+Constant$$

where $l\left(y_{i},{y_{\begin{array}{c} i \end{array}}}^{\left(t-1\right)}+f_{t}\left(x_{i}\right)\right)$ is the loss function, and usually the squared error or the logarithmic loss is used. $\Omega \left(f_{t}\right)$ is the regularization term, which is defined as follows:

(6) $$\Omega \left(f_{t}\right)=\gamma T+\frac{1}{2}\lambda \sum_{j=1}^{T}{{ԝ}^{2}}_{j}$$

where T is the number of leaf nodes. $w_{j}$ is the weight of the j-th leaf node. γ and λ are regularization parameters, which are used to control the complexity of the model.

#### 3.2.4. Lightweight Gradient-Lifting Algorithm (LightGBM)

LightGBM [31] is an efficient gradient-boosting framework. Its core principle is to adopt a decision tree algorithm based on histograms and exclusive feature binding to handle a large number of data instances and a large number of features, which improves the training efficiency and accuracy of the model. Compared with the traditional gradient-boosting decision tree (GBDT) algorithm, LightGBM has higher accuracy and faster training speed.

#### 3.2.5. Evaluation Indicators of Model

The performance of the regression model is evaluated using three indicators: the mean squared error (MSE), the mean absolute error (MAE), and the coefficient of determination (R^2^). The MSE and MAE are indicators for measuring the prediction error [32,33]. Among them, the MSE is the sum of the squares of the differences between the predicted values and the true values, and the MAE is the average of the differences between the predicted values and the true values. The difference between the two is that the former is more sensitive to larger prediction errors, while the latter is more sensitive to smaller prediction errors [34]. The R^2^ value represents the degree of linear correlation between the regression values and the true values, and it is used to measure the extent to which the model can explain the changes in the data. Its value range is from 0 to 1, and a higher R^2^ value indicates that the model can explain the changes in the data better. The formula is as follows:

(7) $$MSE=\frac{1}{m}\sum_{i=1}^{m}(f_{i}-y_{i}{)}^{2}$$

(8) $$MAE=\frac{1}{m}\sum_{i=1}^{n}\left|y_{i}-{\hat{y}}_{i}\right|\;\;$$

(9) $$R^{2}=1-\frac{\sum_{i=0}^{n-1}(y_{i}-f_{i}{)}^{2}}{\sum_{i=0}^{n-1}(y_{i}-{\bar{y}}_{i}{)}^{2}}$$

where m is the number of samples, $f_{i}$ is the true value, and $y_{i}$ is the predicted value

## 4. Conclusions

This research shows that in the prediction of the bandgap and formation energy of double perovskites, the XGBoost model outperforms the GBR, RFR, and LightGBM models. The R^2^ value of the XGBoost model on the double perovskite bandgap test set is 0.934, and the MAE and RMSE are 0.211 eV and 0.259 eV, respectively. When predicting the formation energy, the R^2^ value is 0.959, and the MAE and RMSE are 0.013 eV/atom and 0.091 eV/atom, respectively. The SHAP method is used to interpret the prediction results of the XGBoost model. The analysis shows that the electron affinity of the element at the X position contributes the most to the bandgap prediction and has a positive correlation; the first and third ionization energies of the elements at the B″ position have a negative impact on the bandgap prediction. The first ionization energy of the element at the X position has a negative correlation with the prediction result of the formation energy, and the electronegativities of the elements at the B″ and B′ positions have a relatively large positive correlation with the prediction result of the formation energy. This provides some insights into the hidden relationships between the structure and properties of double perovskites. The element substitution method was adopted to generate 4573 electrically neutral double perovskite structures, and key features were filled to form a prediction dataset. Using the data limitations of the tolerance factor and the octahedral factor, 2054 candidate double perovskite materials with stable structures were screened out as prediction samples. According to the bandgap values predicted by the XGBoost model, 99 lead-free double perovskite candidate materials with a bandgap of 1.3~1.4 eV were screened out. The bandgap range of these candidate materials has the best matching with the solar spectrum, making it possible for them to become ideal materials for photovoltaic absorption layers. In particular, the elemental combinations containing Se/S/O/C (X-site) and Pd/Ir/Fe/Ta/Pt/Cu (B″-site) provide clear guidance for experimental synthesis. The research results provide a rich candidate material library for subsequent research on new perovskite materials, which can significantly reduce the trial-and-error cost of new material development. It will promote the development of non-toxic and highly efficient photovoltaic devices and drive the green and sustainable development of the photovoltaic industry.

In the future, the feature engineering will be further expanded upon to include more structural dynamic parameters (such as lattice vibration, defect states, etc.) to improve the comprehensiveness of the model prediction. Moreover, the 99 lead-free candidate materials selected in this study will be validated by high-throughput experiments to accelerate their practical application.

## Figures and Tables

**Table 1:** Performance of bandgap and formation energy predicted by regression models.

Prediction Target	Regression Model	Evaluation Indicator		
		R^2^	MSE	MAE
Bandgap	XGBoost	0.933	0.218	0.297
	RF	0.909	0.293	0.370
	GBR	0.878	0.393	0.485
	LightGBM	0.931	0.222	0.349
Formation energy	XGBoost	0.948	0.012	0.063
	RF	0.938	0.014	0.074
	GBR	0.939	0.014	0.086
	LightGBM	0.921	0.018	0.086

**Table 2:** Validation results of the XGBoost prediction model on the test set.

Prediction Target	Model	MAE	RMSE	R^2^
Bandgap	XGBoost	0.211	0.259	0.934
Formation Energy	XGBoost	0.013	0.091	0.959

## Data Availability

Data are contained within the article and Supplementary Materials.

## Supplementary Materials

The following supporting information can be downloaded at: https://www.mdpi.com/article/10.3390/molecules30112378/s1, Figure S1: Box plot of the data of 50 feature descriptors. Figure S2: Feature quantity scores of four algorithms in the bandgap dataset. Figure S3: Feature quantity scores of four algorithms in the formation energy dataset. Table S1: Results of ten-fold cross-validation for the bandgap prediction model. Table S2: Results of ten-fold cross-validation for the formation energy prediction model. Table S3: Results of hyperparameter selection for the XGBoost model. Table S4: Lead-free double perovskites with predicted bandgap within the range of 1.3–1.4 eV. Table S5: The 50 descriptors and their physical meanings.
