# Supplementary material for: Prediction and Screening of Lead-Free Double Perovskite Photovoltaic Materials Based on Machine Learning
Source: Molecules. 2025 May 29;30(11):2378. doi: 10.3390/molecules30112378 (PMC12155886; doi:10.3390/molecules30112378)
Supplement: Supplementary file 1 [file molecules-30-02378-s001.zip › supplementary data and dataset/Supplementary data.pdf]

Supplementary data

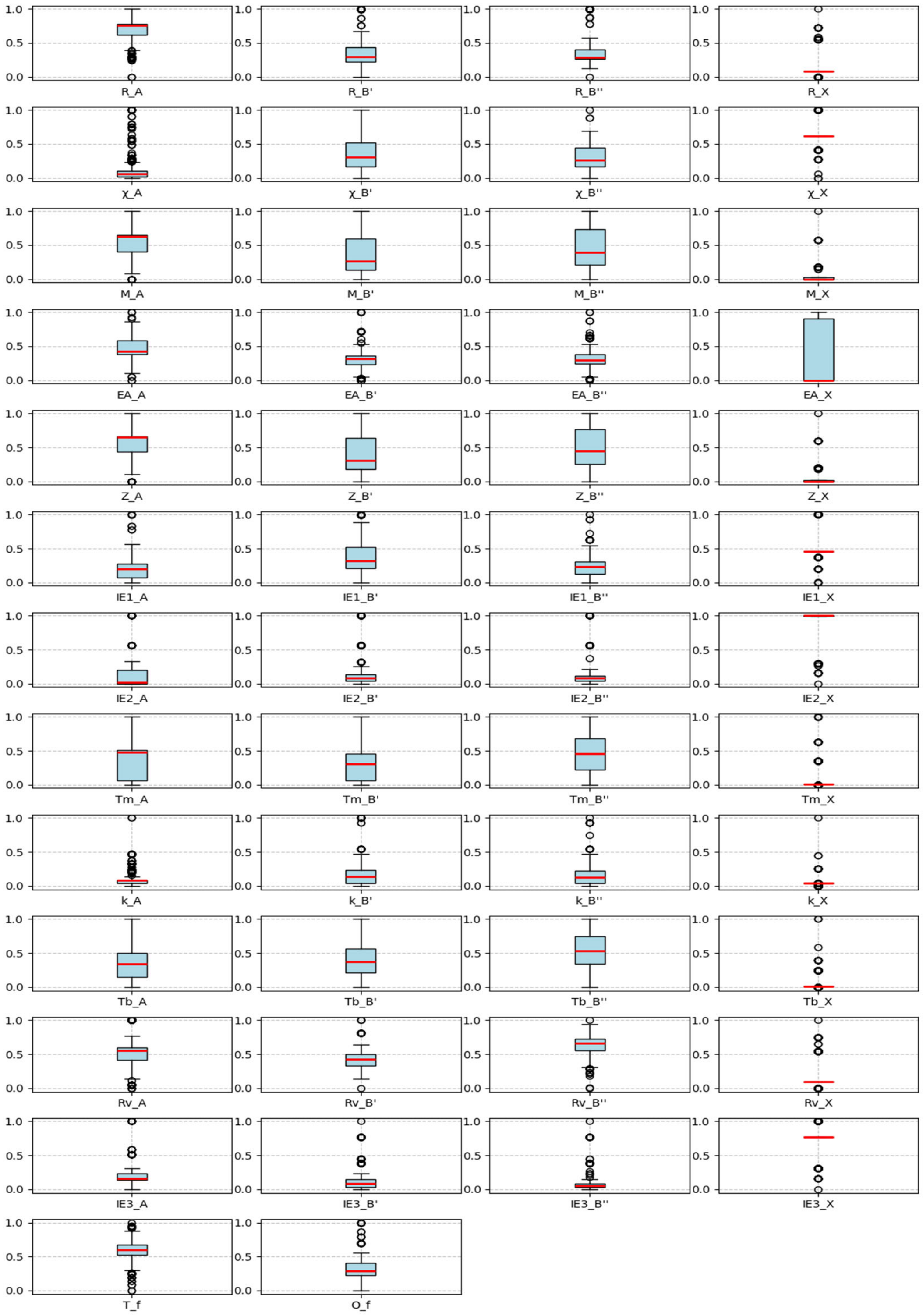

Figure S1 Box plot of the data of 50 feature descriptors

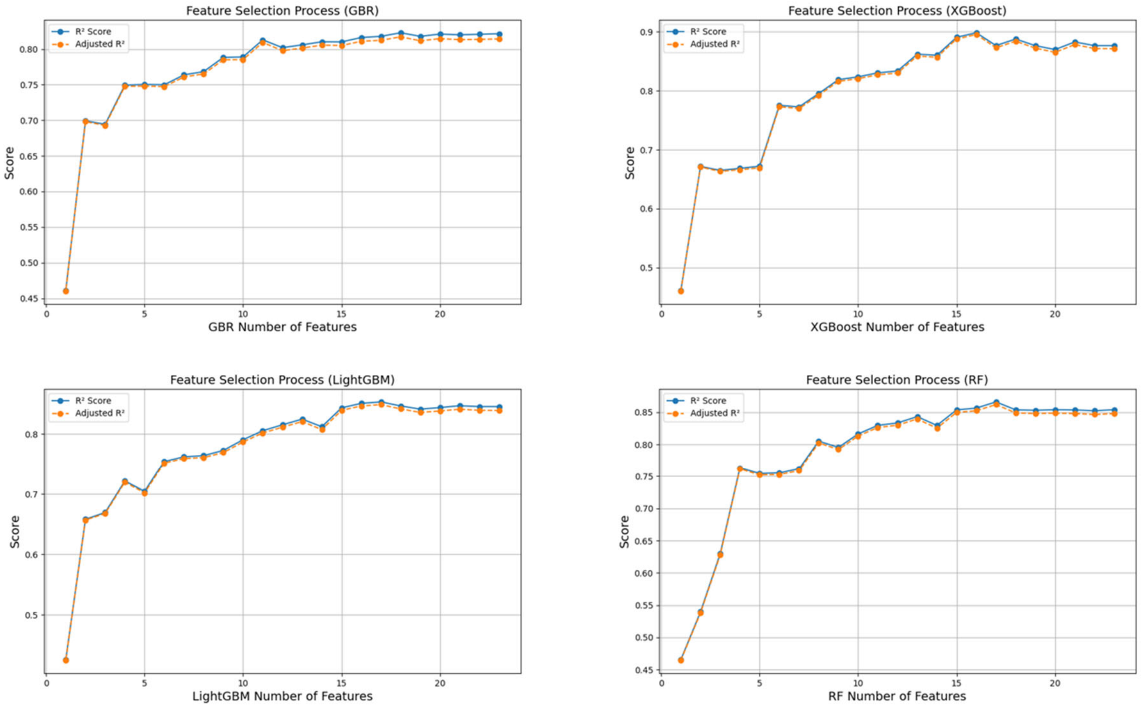

Figure S2 Feature quantity scores of four algorithms in the bandgap dataset

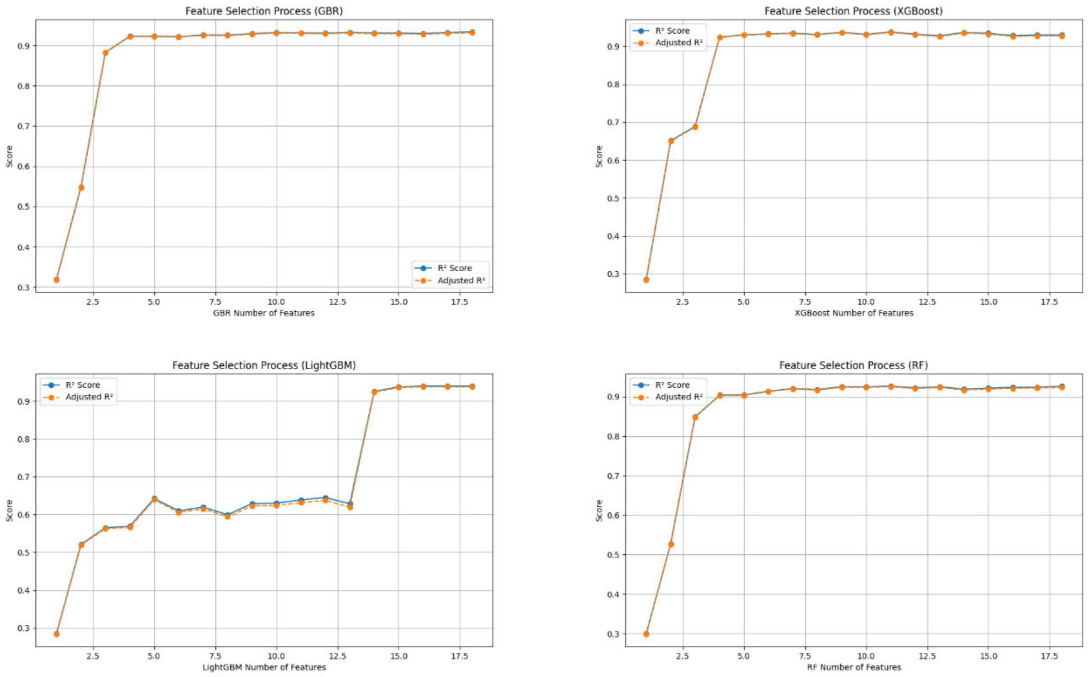

Figure S3 Feature quantity scores of four algorithms in the formation energy dataset

Table S1 Results of ten-fold cross-validation for the bandgap prediction model

Model	Serial Number	R^2	MAE	RMSE
LightGBM	1	0.887456	0.391455	0.582574
	2	0.822929	0.447371	0.616755
	3	0.863722	0.428149	0.591253
	4	0.843649	0.487918	0.684506
	5	0.765688	0.465041	0.69923
	6	0.577631	0.634792	1.118667
	7	0.785869	0.461164	0.750612
	8	0.685448	0.456328	0.816049
	9	0.837408	0.480227	0.681261
	10	0.923552	0.368939	0.508832
RF	1	0.85458	0.419235	0.66222
	2	0.860382	0.389619	0.547657
	3	0.854979	0.415848	0.609924
	4	0.886089	0.395515	0.584266
	5	0.797286	0.43656	0.650377
	6	0.685942	0.578921	0.964628
	7	0.773727	0.456613	0.7716
	8	0.629627	0.431547	0.885503
	9	0.877425	0.434537	0.591512
	10	0.933662	0.340711	0.473992
GBR	1	0.829831	0.543679	0.716358
	2	0.789692	0.555469	0.672151
	3	0.784115	0.542119	0.744169
	4	0.820315	0.544822	0.733809
	5	0.691703	0.603348	0.802061
	6	0.762566	0.594468	0.779883
	7	0.783178	0.57134	0.605875
	8	0.70218	0.535768	0.794049
	9	0.835428	0.577559	0.685395
	10	0.896061	0.481707	0.593307
XGBoost	1	0.879957	0.357374	0.601672
	2	0.833513	0.388559	0.598038
	3	0.868817	0.412847	0.580095
	4	0.895745	0.384602	0.558953
	5	0.810786	0.403746	0.628348
	6	0.812141	0.445737	0.653517
	7	0.830311	0.432862	0.642378
	8	0.842885	0.374756	0.576739
	9	0.933571	0.322033	0.394646
	10	0.918597	0.344005	0.525063

Table S2 Results of ten-fold cross-validation for the formation energy prediction model

Model	Serial Number	R2	MAE	RMSE
LightGBM	1	0.871084	0.0927437	0.1926007
	2	0.9257078	0.1021931	0.1540525
	3	0.9454185	0.0703081	0.1051696
	4	0.9665685	0.0539870	0.0777222
	5	0.9540653	0.0705344	0.1067131
	6	0.9588073	0.066674	0.0954313
	7	0.9183063	0.0920595	0.1440392
	8	0.8864341	0.0875783	0.1701140
	9	0.8961640	0.1016703	0.1793549
	10	0.9547701	0.0733305	0.1076260
RandomForest	1	0.875481	0.086738	0.189288
	2	0.909542	0.097443	0.169989
	3	0.961166	0.061722	0.08871
	4	0.967183	0.052548	0.077005
	5	0.944771	0.074849	0.117012
	6	0.96133	0.065014	0.092462
	7	0.905854	0.087899	0.154628
	8	0.872697	0.093943	0.180109
	9	0.861119	0.11494	0.207425
	10	0.94994	0.066403	0.113227
GradientBoosting	1	0.875034	0.095481	0.189628
	2	0.907409	0.113561	0.171982
	3	0.935447	0.086981	0.114373
	4	0.944166	0.080178	0.100443
	5	0.939262	0.090149	0.122709
	6	0.938796	0.092484	0.116324
	7	0.913009	0.102349	0.148636
	8	0.894624	0.094318	0.163865
	9	0.923795	0.108515	0.15365
	10	0.939961	0.0865	0.124
XGBoost	1	0.841558	0.086559	0.213521
	2	0.894	0.088091	0.184014
	3	0.974585	0.047841	0.071765
	4	0.953891	0.054504	0.091277
	5	0.952878	0.065111	0.108084
	6	0.951009	0.067101	0.104073
	7	0.910378	0.084609	0.150867
	8	0.882431	0.086697	0.173086
	9	0.88598	0.100798	0.187945
	10	0.959051	0.058234	0.102407

Table S3 Results of hyperparameter selection for the XGBoost model

Hyperparameter	Value	
	Predicted Bandgap	Predicted Formation Energy
learning_rate	0.1	0.1
gamma	0	0
colsample_bytree	0.8	0.6
max_depth	6	3
n_estimators	300	300
reg_alpha	0	0
reg_lambda	1	1
subsample	1.0	1.0

Table S4 Lead-free double perovskites with predicted bandgap within the range of 1.3-1.4 eV

Chemical Formula	Predicted Bandgap(eV)	Predicted Formation Energy(eV/atom)	Tolerance Factor	Octahedral Factor
Mg_2VRhO_6	1.391203	-2.99542	0.917728	0.589286
Mg_2VRhS_6	1.391203	-2.92934	0.930891	0.436765
Mg_2VRhSe_6	1.334819	-2.43485	0.934239	0.403533
Mg_2NbFeO_6	1.370094	-3.4763	0.895792	0.621032
Mg_2NbFeS_6	1.370094	-3.38638	0.912128	0.460294
$Mg_2NbFeSe_6$	1.348852	-2.73539	0.916303	0.425272
Mg_2NbAuS_6	1.334898	-2.72102	0.867332	0.520588
$Mg_2NbAuSe_6$	1.323075	-2.32166	0.87334	0.480978
Mg_2CrCoS_6	1.332602	-3.32894	0.895213	0.482353
Mg_2PdWSe_6	1.399595	-2.10635	0.879374	0.472826
Mg_2PdWTe_6	1.332775	-1.94887	0.88778	0.42029
Mg_2PtWTe_6	1.356244	-1.98183	0.899341	0.405797
$Mg_2CuReSe_6$	1.351109	-2.50855	0.921834	0.418478
Ca_2YTaSe_6	1.37357	-2.8365	0.95301	0.494565
Ca_2VAuTe_6	1.370679	-2.25125	0.987428	0.403382
Ca_2NbFeO_6	1.304164	-3.49464	1.014104	0.621032
Ca_2NbFeS_6	1.304164	-3.40472	1.011893	0.460294
Ca_2NbIrO_6	1.381946	-2.90372	1.003608	0.634921
Ca_2NbIrS_6	1.381946	-2.87148	1.003048	0.470588
$Ca_2NbIrSe_6$	1.340569	-2.40531	1.002904	0.434783
Ca_2CrNiO_6	1.386906	-3.33054	1.00808	0.628968
Ca_2CrNiS_6	1.386906	-3.21498	1.006819	0.466176
$Ca_2CrNiSe_6$	1.302631	-2.46414	1.006497	0.430707
$Ca_2CrCuSe_6$	1.386939	-2.47346	0.996973	0.441576
Ca_2MoPdS_6	1.385628	-2.4334	0.971507	0.508824
$Ca_2MoPdSe_6$	1.352228	-2.01395	0.972809	0.470109
$Ca_2MoPdTe_6$	1.398437	-1.88035	0.974708	0.417874
Ca_2FeTaO_6	1.333976	-3.36309	1.014104	0.621032

Chemical Formula	Predicted Bandgap(eV)	Predicted Formation Energy(eV/atom)	Tolerance Factor	Octahedral Factor
Ca_2FeTaS_6	1.333976	-3.26454	1.011893	0.460294
$Ca_2RhTaSe_6$	1.397034	-2.45681	1.006497	0.430707
Ca_2PtWS_6	1.338738	-2.51296	0.9834	0.494118
$Ca_2AgCuCl_6$	1.309419	-3.1805	0.878853	0.646707
$Ca_2AgCuBr_6$	1.309419	-3.1535	0.884281	0.593407
$Ca_2AuTaSe_6$	1.342913	-2.49253	0.963909	0.480978
$La_2ScIrTe_6$	1.368671	-2.36286	0.989737	0.411836
La_2YFeS_6	1.34868	-3.42441	0.960384	0.536765
La_2YFeTe_6	1.361898	-2.692	0.964746	0.440821
La_2YAuS_6	1.384298	-2.70554	0.915984	0.597059
La_2YAuSe_6	1.301994	-2.37038	0.919575	0.55163
La_2ZrCoS_6	1.378961	-3.4061	0.978901	0.513235
$La_2ZrCoSe_6$	1.311509	-2.7802	0.979862	0.474185
$La_2ZrCoTe_6$	1.378281	-2.65661	0.981264	0.421498
La_2ZrPtS_6	1.303529	-2.82007	0.966095	0.529412
$La_2HfCoSe_6$	1.368762	-2.73031	0.982122	0.471467
$La_2HfPtTe_6$	1.376646	-2.282	0.971904	0.432367
La_2CrFeO_6	1.382585	-3.52154	1.035359	0.611111
La_2CrFeS_6	1.382585	-3.40267	1.02978	0.452941
$La_2CrFeSe_6$	1.324282	-2.67233	1.028357	0.418478
La_2FeRhO_6	1.332481	-2.95449	1.020006	0.630952
La_2FeRhS_6	1.332481	-2.8633	1.016889	0.467647
La_2PtMnO_6	1.383153	-3.24884	1.013992	0.638889
La_2PtMnS_6	1.383153	-3.18347	1.011823	0.473529
La_2AuCrS_6	1.320454	-3.06677	0.978901	0.513235
$La_2AuCrSe_6$	1.315185	-2.60291	0.979862	0.474185
$La_2AuCrTe_6$	1.377443	-2.40977	0.981264	0.421498
Bi_2YCrSe_6	1.352344	-2.32523	0.968077	0.487772
Bi_2YIrS_6	1.342082	-2.40166	0.951838	0.547059
$Bi_2TiCoSe_6$	1.326977	-2.03822	1.00582	0.442935
Bi_2ZrMnO_6	1.388618	-2.80914	1.037631	0.607143
Bi_2ZrMnS_6	1.388618	-2.73541	1.031679	0.45
Bi_2HfPdS_6	1.364676	-2.1496	0.954076	0.544118
$Bi_2HfPdSe_6$	1.311618	-1.75211	0.956118	0.502717
$Bi_2HfPdTe_6$	1.344933	-1.64649	0.959106	0.44686
Bi_2VCuSe_6	1.350714	-1.98419	1.025199	0.421196
$Bi_2CrFeSe_6$	1.398722	-2.20455	1.027674	0.418478
Bi_2CrRhO_6	1.327215	-2.38912	1.028316	0.619048
Bi_2CrRhS_6	1.327215	-2.32106	1.023871	0.458824
$Bi_2MnCoSe_6$	1.363011	-1.97799	1.023966	0.422554
$Bi_2FeRhSe_6$	1.391986	-1.88556	1.015417	0.432065
Bi_2FeAuS_6	1.305264	-1.94749	0.971198	0.522059

Chemical Formula	Predicted Bandgap(eV)	Predicted Formation Energy(eV/atom)	Tolerance Factor	Octahedral Factor
Bi_2AgTaS_6	1.314718	-2.66425	0.907162	0.608824
$Na_2NbTaSe_6$	1.354493	-2.63147	1.019338	0.423913
Na_2RuReO_6	1.386036	-2.73158	1.045994	0.59127
Na_2RuReS_6	1.386036	-2.63222	1.038644	0.438235
$Na_2CoNiCl_6$	1.375743	-3.13318	0.981723	0.513473
$Na_2CoNiBr_6$	1.375743	-3.09791	0.982627	0.471154
Na_2CoPdI_6	1.362226	-2.64937	0.949042	0.457524
$Na_2IrCuCl_6$	1.356249	-2.61793	0.98778	0.505988
$Na_2IrCuBr_6$	1.356249	-2.63909	0.988388	0.464286
$Na_2IrAgCl_6$	1.389706	-2.4599	0.895004	0.631737
$Na_2IrAgBr_6$	1.389706	-2.48105	0.89976	0.57967
Na_2NiCoI_6	1.39913	-3.25267	0.983901	0.416262
Na_2PdNiI_6	1.310841	-2.69386	0.960046	0.444175
Na_2PdPtI_6	1.362329	-2.0893	0.938287	0.470874
Na_2PdCuI_6	1.302642	-2.61254	0.952018	0.453883
Na_2PdCdI_6	1.362664	-2.55998	0.910156	0.507282
Na_2PtCdI_6	1.396732	-2.59983	0.921204	0.492718
Na_2CuRhI_6	1.372131	-2.82765	0.992478	0.406553
Na_2CuPdI_6	1.399947	-2.76575	0.952018	0.453883
$Na_2AuReSe_6$	1.36013	-2.3562	0.995422	0.451087
$Na_2AuReTe_6$	1.317659	-2.22245	0.995747	0.400966
Cs_2YAgBr_6	1.368234	-2.85944	1.046846	0.64011
Sr_2ScTaS_6	1.392586	-3.33452	1.049583	0.489706
$Sr_2CrPtSe_6$	1.333473	-2.10509	1.039971	0.460598
Sr_2CuAgI_6	1.374313	-3.08031	0.9422	0.524272
Sr_2AuVSe_6	1.398499	-2.72554	1.046057	0.453804
$Sr_2AuTaTe_6$	1.373461	-2.313	1.020599	0.427536
K_2PdPtI_6	1.365693	-2.10089	1.043188	0.470874
K_2PdCdI_6	1.364544	-2.57499	1.011913	0.507282

Table S5 The 50 descriptors and their physical meanings

Number	Feature Name	Description	Unit
1	R_A	A - site Ionic Radius	Å
2	R_B'	B'-site Ionic Radius	Å
3	R_B''	B''-site Ionic Radius	Å
4	R_X	X-site Ionic Radius	Å
5	χ_A	Electronegativity of A-site Atom	eV
6	χ_B'	Electronegativity of B'-site Atom	eV
7	χ_B''	Electronegativity of B''-site Atom	eV
8	χ_X	Electronegativity of X - site Atom	eV
9	M_A	Atomic mass of A - site element	g/mol
10	M_B'	Atomic mass of B'-site element	g/mol
11	M_B''	Atomic mass of B''-site element	g/mol
12	M_X	Atomic mass of X - site element	g/mol
13	EA_A	Electronic affinity of A - site element	eV
14	EA_B'	Electronic affinity of B'-site element	eV
15	EA_B''	Electronic affinity of B''-site element	eV
16	EA_ X	Electronic affinity of X - site element	eV
17	Z_A	Atomic number of A - site element	dimensionless
18	Z_B'	Atomic number of B'-site element	dimensionless
19	Z_B''	Atomic number of B''-site element	dimensionless
20	Z_X	Atomic number of X - site element	dimensionless
21	IE1_A	The first ionization energy of the A - site element	eV
22	IE1_B'	The first ionization energy of the B'-site element	eV
23	IE1_B''	The first ionization energy of the B''-site element	eV
24	IE1_X	The first ionization energy of the X - site element	eV
25	IE2_A	The second ionization energy of the A - site element	eV
26	IE2_B'	The second ionization energy of the B' - site element	eV
27	IE2_B''	The second ionization energy of the B''-site element	eV
28	IE2_X	The second ionization energy of the X - site element	eV
29	IE3_A	The third ionization energy of the A - site element	eV
30	IE3_B'	The third ionization energy of the B'-site element	eV
31	IE3_B''	The third ionization energy of the B''-site element	eV
32	IE3_X	The third ionization energy of the X - site element	eV
33	Tm_A	The melting point temperature of the A - site element	K
34	Tm_B'	The melting point temperature of the B'-site element	K
35	Tm_B''	The melting point temperature of the B''-site element	K
36	Tm_X	The melting point temperature of the X - site element	K
37	k_A	The thermal conductivity of the A - site element	W/(m*k)
38	k_B'	The thermal conductivity of the B'-site element	W/(m*k)
39	k_B''	The thermal conductivity of the B''-site element	W/(m*k)
40	k_X	The thermal conductivity of the X-site element	W/(m*k)
41	Tb_A	The boiling point temperature of the A-site element	K

Number	Feature Name	Description	Unit
42	Tb_B'	The boiling point temperature of the B'-site element	K
43	Tb_B''	The boiling point temperature of the B''-site element	K
44	Tb_X	The boiling point temperature of the X - site element	K
45	Rv_A	The van der Waals radius of the A - site element	Å
46	Rv_B'	The van der Waals radius of the B'-site element	Å
47	Rv_B''	The van der Waals radius of the B''-site element	Å
48	Rv_X	The van der Waals radius of the X - site element	Å
49	T_f	Tolerance factor	dimensionless
50	O_f	Octahedral factor	dimensionless
